# Effects of exergaming on balance and functional performance in older adults with chronic conditions living in the community: a systematic review and meta-analysis

**DOI:** 10.3389/fpubh.2026.1918722

**Published:** 2026-09-07

**Authors:** Beihai Ge, Jinsong Luo, Xiaofeng Cheng, Deng Wang, Renming Liu, Xianhui Liao

**Affiliations:** 1 Rugao People’s Hospital Affiliated to Kangda College of Nanjing Medical University, Nantong, Jiangsu, China; 2 Wuchang University of Technology, Wuhan, Hubei, China; 3 Department of Infectious Diseases, Shangyu People's Hospital of Shaoxing, Shaoxing University, Shaoxing, China; 4 School of Physical Education, Guangzhou University of Sport, Guangzhou, China; 5 Neuromodulation Research Unit, Department of Physiotherapy, School of Primary and Allied Health Care, Monash University, Melbourne, VIC, Australia; 6 Department of physiotherapy, School of Primary and Allied Health Care, Monash University, Melbourne, VIC, Australia

**Keywords:** balance, chronic disease, exergaming, functional performance, meta-analysis, older adults, rehabilitation

## Abstract

**Background:**

Older adults with chronic diseases often experience impaired balance and functional performance, increasing their risk of falls and disability. Exergaming has emerged as a promising intervention; however, its effectiveness in community-dwelling older adults with chronic conditions remains unclear.

**Objective:**

To systematically review and meta-analyze the effects of exergaming on balance and functional performance in community-dwelling older adults with chronic diseases.

**Methods:**

Following PRISMA guidelines, PubMed, Embase, Web of Science, Cochrane Library, Scopus, and CINAHL were searched from inception to April 10, 2026. Randomized controlled trials involving exergaming interventions in adults aged ≥60 years with chronic conditions were included. Primary outcomes were balance and functional mobility. Risk of bias was assessed using the Cochrane Risk of Bias 2 tool. Pooled effects were calculated using mean differences (MDs) or standardized mean differences (SMD) with 95% confidence intervals (CIs) according to outcome measurement scales.

**Results:**

Eleven randomized controlled trials were included. Compared with control interventions, exergaming significantly improved balance measured by the Berg Balance Scale (MD = 2.93, 95% CI 0.44–5.41, *p* = 0.021) and dynamic gait ability measured by the Dynamic Gait Index (MD = 0.72, 95% CI 0.09–1.35, *p* = 0.025). Subgroup analyses indicated greater benefits for interventions lasting at least 8 weeks and those involving supervised training. Heterogeneity was low to moderate.

**Conclusion:**

Exergaming may be a promising adjunctive approach for improving balance and functional performance in community-dwelling older adults with chronic diseases. Further high-quality trials with larger sample sizes and long-term follow-up are needed.

**Systematic review registration:**

PROSPERO registration number: CRD420261368359.

## Introduction

Population aging has become a major global public health challenge, accompanied by a rapidly increasing prevalence of chronic diseases among older adults (1, 2). Chronic conditions such as stroke, Parkinson’s disease, diabetes mellitus, osteoarthritis, and cardiovascular disease are frequently associated with impairments in balance, gait, muscle strength, and functional mobility (3). These impairments substantially increase the risks of falls, disability, hospitalization, institutionalization, and reduced quality of life among older adults (4). Falls are one of the leading causes of injury, functional decline, and loss of independence in the older adults’ population, imposing considerable physical, psychological, and economic burdens on individuals, families, and healthcare systems (5, 6). Consequently, the development of effective, safe, and accessible rehabilitation strategies to improve balance and functional performance in community-dwelling older adults with chronic diseases has become an important public health priority (7).

Exercise-based rehabilitation is widely recommended for maintaining physical function, enhancing mobility, and reducing fall risk in older adults with chronic conditions (8). However, adherence to conventional exercise programs is often limited because of reduced motivation, monotony, transportation barriers, and concerns regarding safety or accessibility (9, 10). In recent years, exergaming has emerged as an innovative rehabilitation approach that combines physical activity with interactive virtual gaming environments (11, 12). In rehabilitation settings, exergaming specifically refers to exercise-based interventions that require participants to perform purposeful physical movements through interactive digital platforms, rather than general recreational gaming activities. Exergaming interventions commonly integrate task-oriented movements, multisensory visual and auditory feedback, cognitive engagement, motor learning principles, and real-time interaction, thereby providing a more engaging and motivating exercise experience than traditional rehabilitation programs (13, 14). These features may improve exercise adherence, participation, and long-term training sustainability among older adults (15, 16).

Previous studies have suggested that exercise-based exergaming may improve balance performance, gait ability, lower-extremity muscle strength, postural control, and functional mobility in both healthy older adults and clinical populations (17, 18). For example, interactive exergaming platforms, such as Nintendo Wii-based systems, have been applied in older adults to provide task-oriented balance training and real-time feedback, while motion-sensing technologies such as Microsoft Kinect allow users to perform whole-body movements through virtual environments, potentially enhancing engagement and functional exercise participation (17, 18). Moreover, commercially available platforms such as Nintendo Wii and Microsoft Kinect have increased the accessibility and feasibility of exercise-based exergaming in home- and community-based settings (12, 16). These platforms enable participants to engage in repetitive functional movements, including balanced tasks, stepping activities, and simulated daily activities, which may facilitate continued participation in exercise programs among older adults. Given their relatively low cost, portability, and ease of implementation, exercise-based exergaming may represent a promising strategy for long-term rehabilitation and self-management in older adults with chronic diseases living in the community (15, 19).

Despite the growing interest in exergaming, the current evidence regarding its effectiveness in community-dwelling older adults with chronic diseases remains limited and inconsistent (20, 21). Existing systematic reviews have primarily focused on healthy older adults, institutionalized populations, or patients with specific neurological disorders, while evidence involving older adults with diverse chronic conditions living in community settings has not been comprehensively synthesized (22, 23). In addition, substantial variations exist across studies in terms of intervention duration, training frequency, supervision, exercise intensity, and gaming platforms, all of which may influence rehabilitation outcomes (24). The heterogeneity of chronic disease characteristics and intervention protocols further complicates the interpretation of current findings (25). Therefore, a focused systematic review and meta-analysis is needed to quantitatively evaluate the effects of exercise-based exergaming on balance and functional performance in this population.

Accordingly, the aim of this systematic review and meta-analysis was to evaluate the effects of exergaming interventions on balance and functional performance in community-dwelling older adults with chronic diseases. Furthermore, subgroup analyses were conducted to examine the potential influences of intervention duration and supervision on rehabilitation outcomes. The findings of this study may provide evidence-based support for the integration of exercise-based exergaming into community rehabilitation programs and chronic disease management strategies for older adults (26).

## Methods

This systematic review and meta-analysis were conducted in accordance with the Preferred Reporting Items for Systematic Reviews and Meta-Analyses (PRISMA) guidelines (27). The review protocol was prospectively registered in the International Prospective Register of Systematic Reviews (PROSPERO; registration number: CRD420261368359) (28).

### Eligibility criteria

The eligibility criteria were established according to the PICOS framework (Participants, Intervention, Comparison, Outcomes, and Study design).

This study included older adults aged 60 years and older residing in the community with chronic diseases. Eligible chronic diseases included, but were not limited to, stroke, Parkinson’s disease, diabetes, osteoarthritis, cardiovascular disease, and other chronic diseases associated with impaired balance or functional mobility. Studies involving older adults residing in nursing homes, hospitals, or long-term care facilities were excluded. Eligible studies investigated motion-sensing game-based interventions, including interactive video game exercise programs offered through platforms such as Nintendo Wii, Wii Fit, Microsoft Kinect, virtual reality systems, immersive or non-immersive VR technologies, and other comparable interactive motion-based gaming platforms involving body movement detection, gesture recognition, or real-time movement feedback. Interventions could be supervised or unsupervised and could be conducted in a community or home setting. Control interventions included routine care, health education, waitlist controls, no intervention, or traditional non-motion-sensing game interventions. Studies must report at least one outcome related to balance, mobility, function, or falls. Primary outcome measures included: Balance (Berg Balance Scale [BBS], Postural Control Indicators); Functional activity levels (Timed Stand-Up-Walk Test [TUG], Dynamic Gait Index [DGI]). Secondary outcome measures included: Gait performance (e.g., gait speed, 6-min walk test [6MWT]); Lower limb muscle strength; Functional motor ability; Activities of daily living.

This study only included randomized controlled trials (RCTs) published in English. Observational studies, quasi-experimental studies, conference abstracts, reviews, study protocols, and studies with insufficient data to calculate effect sizes were excluded (Supplementary S1).

### Information sources and search strategy

We conducted a comprehensive literature search in PubMed, Embase, Cochrane Library, CINAHL, and Web of Science databases, covering the period from database inception to April 10, 2026. The search strategy combined controlled terminology (MeSH and Emtree terms) with free-text keywords related to motion-sensing games, older adults, chronic diseases, balance, functional activity levels, and randomized controlled trials.

Search terms related to motion-sensing games included “motion-sensing games,” “interactive video games,” “virtual reality,” “Nintendo Wii,” “Wii Fit,” “Microsoft Kinect,” and other related motion-sensing game technologies. Search terms related to older adults included “older adults,” “elderly,” “senior citizens,” and “community residents.” Search terms related to chronic diseases included “stroke,” “Parkinson’s disease,” “diabetes,” “osteoarthritis,” “cardiovascular disease,” and “chronic diseases.” Search terms related to outcomes included “balance,” “postural control,” “Berg Balance Scale,” “timed stand-up walk test,” “gait,” “walking speed,” “6-min walk test,” and other functional activity assessment indicators. Randomized controlled trials were screened where necessary. The final search results showed 404 records in PubMed, 325 in Embase, 244 in the Cochrane Library, 89 in CINAHL, and 147 in Web of Science. In addition, we manually screened the references of the included studies and related systematic reviews to identify potentially qualified studies (S4).

### Data management, screening and selection, and extraction

Throughout the review process, we used Covidence, Endnote, and Excel applications to manage records (29). The removal of duplicate references, deletion of titles and abstracts, and full-text review were all documented using PRISMA flowcharts (27).

References were imported into the Covidence system for duplicate removal. Two reviewers independently screened titles and abstracts according to inclusion and exclusion criteria. Disagreements were resolved through discussion; if a consensus could not be reached, a third reviewer made the final decision. The two reviewers independently extracted data using standardized data extraction forms. If data was insufficient or unclear, researchers contacted the study authors to obtain missing information. Commonly extracted study characteristics included: first author, publication year, language, country/region, sample characteristics (sample size, mean age and/or age range), intervention characteristics (dose [frequency, duration, administration time]), control characteristics (active or inactive control), and assessment tools used. Disagreements were resolved through discussion; if a consensus could not be reached, a third reviewer made the final decision.

### Risk of bias in individual studies

Two reviewers used the Cochrane Risk of Bias 2.0 tool (RoB 2.0) (30) to assess the risk of bias of the included trials at the research level. For each trial that met the inclusion criteria, we assessed its risk of bias in five aspects: randomization process, bias due to deviations from intended interventions, missing outcome data, bias in measurement of the outcome, and bias in selection of the reported result. For each aspect, we evaluated the specific study based on the signaling issues listed in RoB 2.0 and determined the risk of bias to be low risk of bias, some concerns, or high risk of bias. If there were any disagreements among the reviewers, they were resolved through discussion; if necessary, a third reviewer was involved to reach a consensus.

### Data synthesis

All meta-analyses were performed using STATA 18.0 (31). Meta-analyses were used when three or more studies reported the same balance function or fall-related measure. We used random-effects models with the Der Simonian–Laird estimator to estimate between-study variance to calculate the mean difference (MD) or standardized mean difference (SMD) (32, 33) and the corresponding 95% confidence intervals (34, 35). MD was used when studies reported the same outcome measure using the same measurement scale, whereas SMD was applied when the same outcome was assessed using different measurement instruments or scales. *I*^2^ values were calculated and rated according to the following criteria: low (<25%), moderate (25–50%), substantial (50–75%), and considerable (>75%) heterogeneity (36, 37). Statistical significance was defined as a two-sided *p* value <0.05. Results were presented in the form of forest plots and publication bias for each outcome was assessed using the Egger test (38). When there were at least 10 studies available for an outcome, a funnel plot was used for a visual assessment of publication bias. Due to the limited number of studies included in each meta-analysis, sensitivity analyses were not performed because the results would have limited interpretability.

### Subgroup analysis

To explore potential sources of heterogeneity, we conducted subgroup analyses based on the type of motion-sensing gaming platform. The study was divided into Wii Fit-based and Kinect Sports-based interventions. We calculated the pooled effect estimate for each subgroup using a random-effects model. Differences between subgroups were assessed using subgroup difference tests, with a statistical significance level set at *p* < 0.05.

## Results

### Study selection

**Figure 1:**
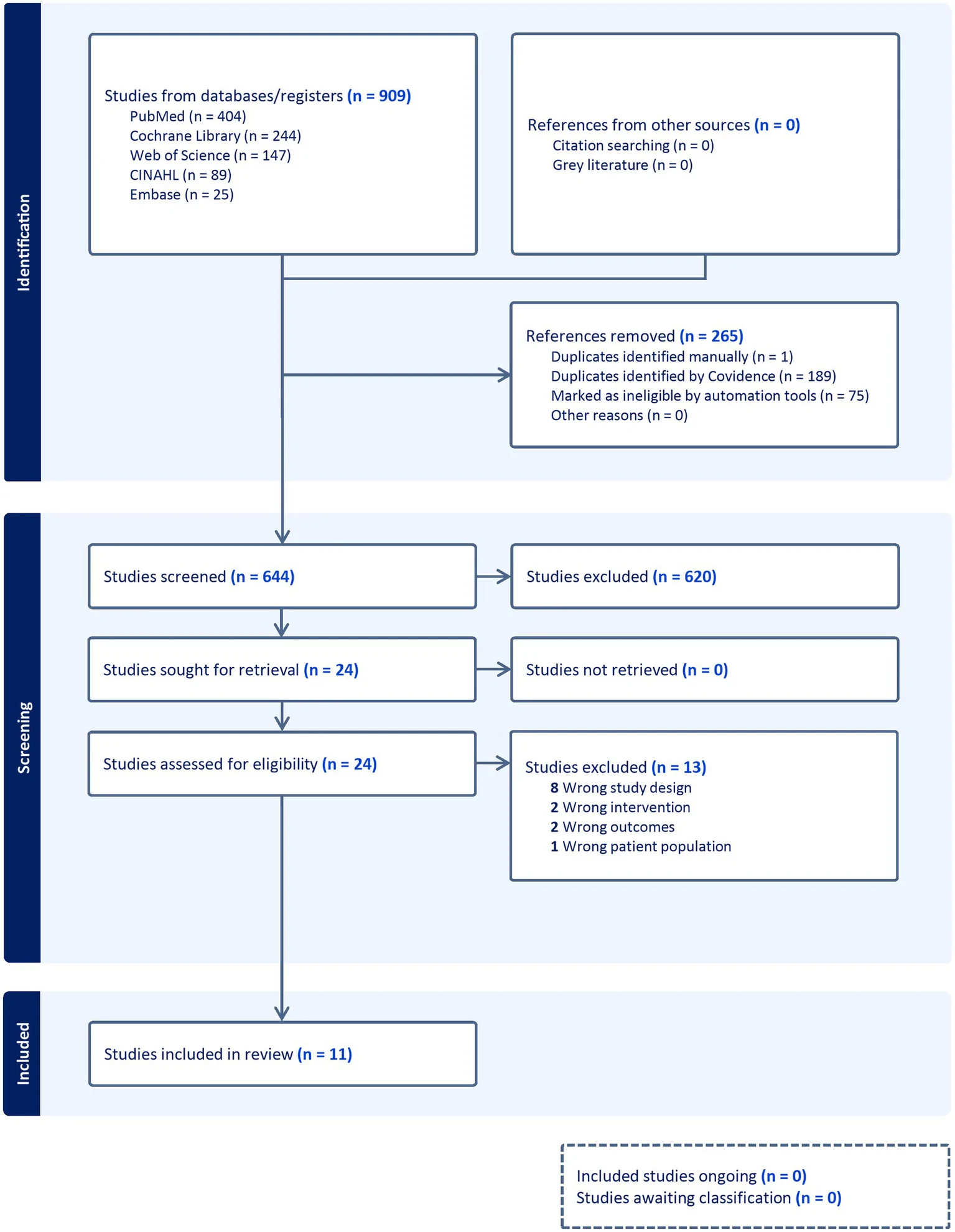
Flowchart of literature screening and inclusion for systematic review and meta-analysis (57).

A total of 1,209 records were found in the database search, including 404 in PubMed, 325 in Embase, 244 in the Cochrane Library, 89 in CINAHL, and 147 in Web of Science. After removing duplicate records, the remaining 644 studies underwent title and abstract screening. Following screening, 24 articles were considered potentially eligible for inclusion and underwent full review. Of these, 13 studies were excluded due to reasons such as not meeting inclusion criteria, non-motion-based game interventions, inappropriate study design, or insufficient outcome data. Ultimately, 11 randomized controlled trials met the inclusion criteria and were included in the systematic review and meta-analysis (39–49). The detailed study selection process is illustrated in the PRISMA flow diagram (Figure 1).

### Study characteristics

This systematic review and meta-analysis included 11 randomized controlled trials involving 442 participants. Sample sizes ranged from 20 to 73 participants. The weighted mean age of the participants was 67.2 years. Gender information was available for 413 participants (118 males and 295 females); gender information was missing for 29 participants (see Table 1).

**Table 1:** General participant and study characteristics.

Characteristics		Number	Mean
Total sample size		442 (100)	
	Age, years		67.15
Sex	Male participants, *n* (%)	118 (26.69)*	
	Female participants, *n* (%)	296 (66.96)*	
Country			
	China	4 (36.4)	
	Brazil	2 (18.2)	
	Turkey	1 (9.1)	
	Malaysia	1 (9.1)	
	United States	1 (9.1)	
	Spain	1 (9.1)	
	Italy	1 (9.1)	
Language			
	English	11 (100)	

*Singh et al (2013, English, Malaysia) (48) data on age and sex not reported.

General participant and study characteristics (*n* = 28).

The studies included came from seven countries. China had the most studies (36.4%), followed by Brazil (18.2%). The remaining studies were from Turkey, Malaysia, the United States, Spain, and Italy (9.1%). Overall, more than half of the included studies were conducted in Asia (54.5%), followed by South America (18.2%), Europe (18.2%), and North America (9.1%). Participants were older adults living in the community with multiple chronic diseases, including stroke, Parkinson’s disease, diabetes, and other chronic conditions associated with balance and mobility impairments. All studies have explored interventions based on motion-sensing games delivered through interactive gaming platforms. Six studies used the Nintendo Wii Fit system, and five used Microsoft Kinect Sports-based interventions (Table 2). Interventions lasted 4 to 16 weeks, with training frequency ranging from 2 to 5 times per week. Most interventions were performed 2 to 3 times per week, with each session lasting 30 to 60 min. Control groups received routine care, health education, or traditional exercise programs (Table 3). The most frequently reported outcome measures included the Berg Balance Scale (50), the Timed Stand-Up Walk Test (TUG), the Dynamic Gait Index (DGI), the Functional Forward Extension Test (FRT), the 6-Minute Walk Test (6MWT), and other measures of balance and functional mobility (Table 4).

**Table 2:** Characteristics of the included studies.

First author (year, language, location)	Sample characteristics			
	Intervention/control	Sample size	Sex	Age
Chan et al. (2024, English, China) (41)	T: Wii Fit	21	Male/female: 6/15	68.2 ± 6.0
	C: usual care	21	Male/female: 4/17	71.4 ± 5.1
Ribas et al. (2017, English, Brazil) (47)	T: Wii Fit	10	Male/female: 4/6	61.70 ± 6.83
	C: usual care	10	Male/female: 4/6	60.20 ± 11.29
Karasu et al. (2018, English, Turkey) (44)	T: Wii Fit	12	Male/female: 5/7	62.3 ± 11.79
	C: usual care	11	Male/female: 5/6	64.1 ± 12.2
Yu et al. (2020, English, China) (49)	T: Kinect Sports	21	Male/female: 5/16	64.25 ± 4.75
	C: usual care	22	Male/female: 3/19	63.75 ± 4.23
Bacha et al. (2018, English, Brazil) (39)	T: Kinect Sports	23	Male/female: 8/15	71.0 (66.0; 74.5)
	C: usual care	23	Male/female: 4/19	66.5 (65.0; 71.75)
Huang et al. (2025, English, China) (43)	T: Kinect Sports	30	Male/female: 5/25	69.10 ± 5.76
	C: usual care	30	Male/female: 6/24	68.70 ± 4.31
Singh et al. (2013, English, Malaysia) (48)	T: Kinect Sports	15	Male/female: NR	65.4 ± 9.8
	C: usual care	13	Male/female: NR	67.0 ± 8.4
Lee et al. (2017, English, China) (45)	T: Kinect Sports	26	Male/female: 16/10	59.35 ± 8.95
	C: usual care	21	Male/female: 18/3	55.76 ± 9.59
Franco et al. (2012, English, America) (42)	T: Wii Fit	11	Male/female: 2/9	79.8 ± 4.7
	C: usual care	11	Male/female: 2/9	76.9 ± 6.3
Carcelén-Fraile et al. (2024, English, Spain) (40)	T: Wii Fit	37	Male/female: 12/25	69.89 ± 1.96
	C: usual care	36	Male/female: 9/27	70.42 ± 1.52
Morone et al. (2016, English, Italy) (46)	T: Wii Fit	19	Male/female: 0/19	67.8 ± 2.98
	C: usual care	19	Male/female: 0/19	70.05 ± 4.93

I, Intervention group; C, Control group; NR, (Not report).

**Table 3:** Dosage of traditional chinese exercises exercise program.

First author (year, language, location)	Dosage			
	Intervention/control	Duration (weeks)	Frequency (per week)	Intensity (min)
Chan et al. (2024, English, China) (41)	T: Wii Fit	16	2	60
	C: usual care	NR	NR	NR
Ribas et al. (2017, English, Brazil) (47)	T: Wii Fit	12	2	30
	C: usual care	NR	NR	NR
Karasu et al. (2018, English, Turkey) (44)	T: Wii Fit	4	5	20
	C: usual care	NR	NR	NR
Yu et al. (2020, English, China) (49)	T: Kinect Sports	10	3	50
	C: usual care	NR	NR	NR
Bacha et al. (2018, English, Brazil) (39)	T: Kinect Sports	12	2	60
	C: usual care	NR	NR	NR
Huang et al. (2025, English, China) (43)	T: Kinect Sports	8	3	30
	C: usual care	NR	NR	NR
Singh et al. (2013, English, Malaysia) (48)	T: Kinect Sports	6	2	120
	C: usual care	NR	NR	NR
Lee et al. (2017, English, China) (45)	T: Kinect Sports	6	2	90
	C: usual care	NR	NR	NR
Franco et al. (2012, English, America) (42)	T: Wii Fit	10	2	30
	C: usual care	NR	NR	NR
Carcelén-Fraile et al. (2024, English, Spain) (40)	T: Wii Fit	12	2	30
	C: usual care	NR	NR	NR
Morone et al. (2016, English, Italy) (46)	T: Wii Fit	8	2	60
	C: usual care	NR	NR	NR

I, Intervention group; C, Control group; NR, (Not report).

**Table 4:** Characteristics of the included studies.

First author (year, language, location)	Intervention/control	Outcome	Adverse events (Yes/No/No report)	Follow-up (Yes/No/No report)
Chan et al. (2024, English, China) (41)	T: Wii Fit	TUG, Dynamic Gait Index	No report	No report
	C: usual care	TUG, Dynamic Gait Index	No report	No report
Ribas et al. (2017, English, Brazil) (47)	T: Wii Fit	BBS, 6MWT	No	Yes
	C: usual care	BBS, 6MWT	No	Yes
Karasu et al. (2018, English, Turkey) (44)	T: Wii Fit	BBS, TUG, FRT	No	Yes
	C: usual care	BBS, TUG, FRT	No	Yes
Yu et al. (2020, English, China) (49)	T: Kinect Sports	Dynamic balance	Drop out: (*n* = 1)	Yes
	C: usual care	Dynamic balance	Drop out: (*n* = 2)	Yes
Bacha et al. (2018, English, Brazil) (39)	T: Kinect Sports	Functional Gait Assessment (FGA), 6MST, Mini-Balance Evaluation Systems Test (Mini-BESTest)	No	Yes
	C: usual care	Functional Gait Assessment (FGA), 6MST, Mini-Balance Evaluation Systems Test (Mini-BESTest)	No	Yes
Huang et al. (2025, English, China) (43)	T: Kinect Sports	6MWT	Drop out: (*n* = 2)	Yes
	C: usual care	6MWT	Drop out: (*n* = 3)	Yes
Singh et al. (2013, English, Malaysia) (48)	T: Kinect Sports	TUG, 6MWT	Drop out: (*n* = 7)	Yes
	C: usual care	TUG, 6MWT	No	Yes
Lee et al. (2017, English, China) (45)	T: Kinect Sports	BBS, TUG, FRT	Drop out: (*n* = 3)	Yes
	C: usual care	BBS, TUG, FRT	Drop out: (*n* = 1)	Yes
Franco et al. (2012, English, America) (42)	T: Wii Fit	BBS, Dynamic Gait Index, Dynamic balance	No report	No report
	C: usual care	BBS, Dynamic Gait Index, Dynamic balance	No report	No report
Carcelén-Fraile et al. (2024, English, Spain) (40)	T: Wii Fit	Dynamic Gait Index	Drop out: (*n* = 1)	Yes
	C: usual care	Dynamic Gait Index	Drop out: (*n* = 2)	Yes
Morone et al. (2016, English, Italy) (46)	T: Wii Fit	BBS	No	Yes
	C: usual care	BBS	No	Yes

BBS, Berg Balance Scale; TUG, Timed Up and Go Test; FRT, Functional Reach Test; 6MWT, 6-Minute Walk Test; DGI, Dynamic Gait Index; FGA, Functional Gait Assessment; 6MST, 6-Minute Step Test; Mini-BESTest, Mini-Balance Evaluation Systems Test.

### Effects of exergaming on balance and functional performance

**Figure 2:**
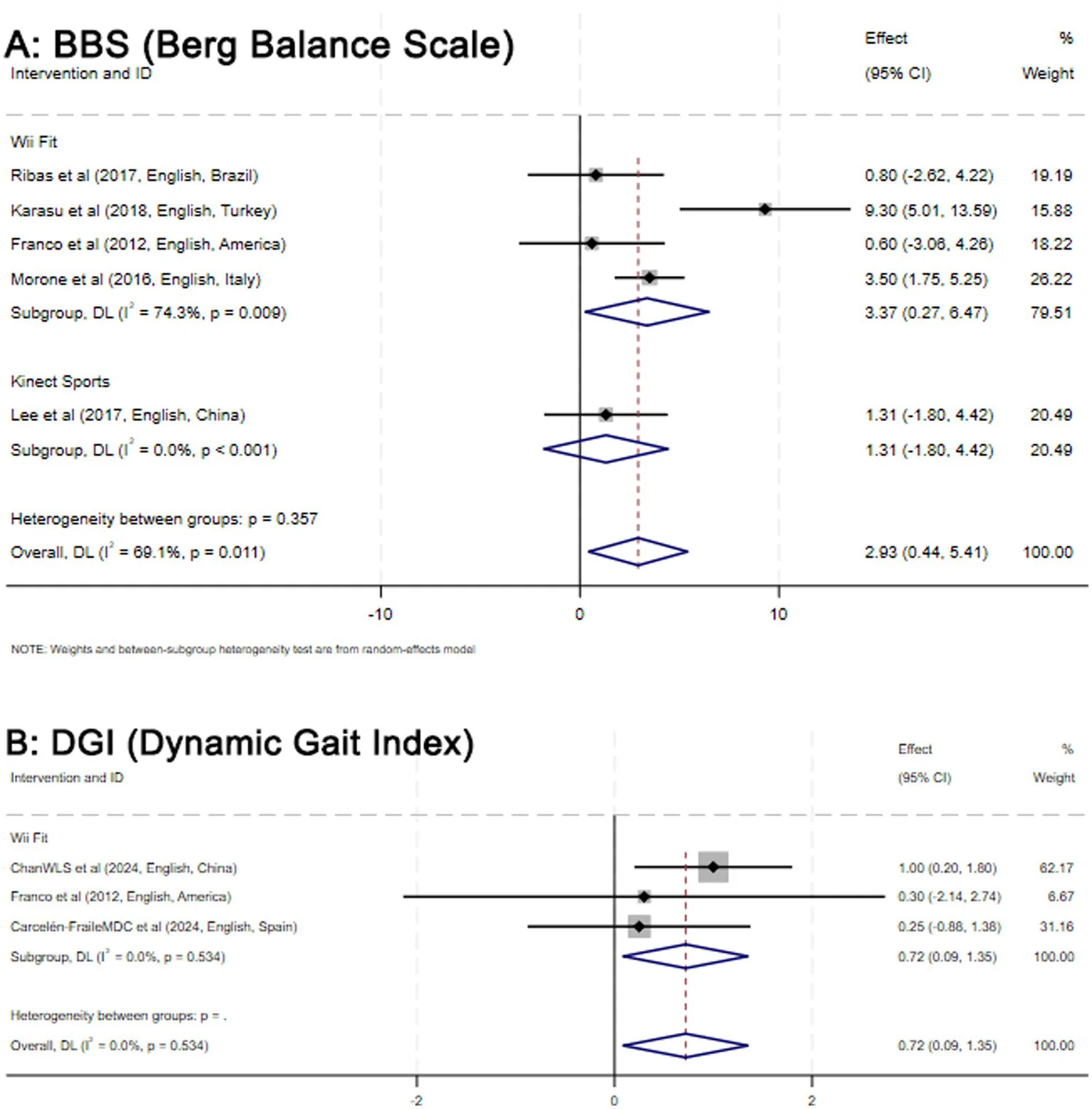
The impact of Exergaming on balance and performance metrics. **(A)** (BBS Beg balance scale); **(B)** DGI (Dynamic Gait index).

Five studies assessed balance using the Berg Balance Scale (50). Meta-analysis showed that motion-sensing games significantly improved balance compared to the control intervention (MD = 2.93, 95% CI 0.44–5.41, *p* = 0.021). Moderate to high heterogeneity was observed among studies (*I*^2^ = 69.1%, *p* = 0.011) (see Figure 2A).

Subgroup analyses based on the motion-sensing game platforms showed that the Wii Fit-based intervention significantly improved BBS scores (MD = 3.37, 95% CI 0.27–6.47), while the Kinect Sports-based intervention did not show a statistically significant effect (MD = 1.31, 95% CI − 1.80 to 4.42). However, subgroup differences did not reach statistical significance (*p* = 0.357), indicating no significant difference in therapeutic effect among different motion-sensing game platforms. Three studies used the Dynamic Gait Index (DGI) to assess dynamic gait ability. A pooled analysis showed that motion-sensing games significantly improved DGI scores compared to the control intervention (MD = 0.72, 95% CI 0.09–1.35, *p* = 0.025). No significant heterogeneity was observed among the studies (*I*^2^ = 0.0%, *p* = 0.534) (see Figure 2B). All studies included in this analysis used Wii Fit–based interventions; therefore, subgroup comparisons according to exergaming platform were not conducted.

### Risk of bias and publication bias

**Figure 3:**
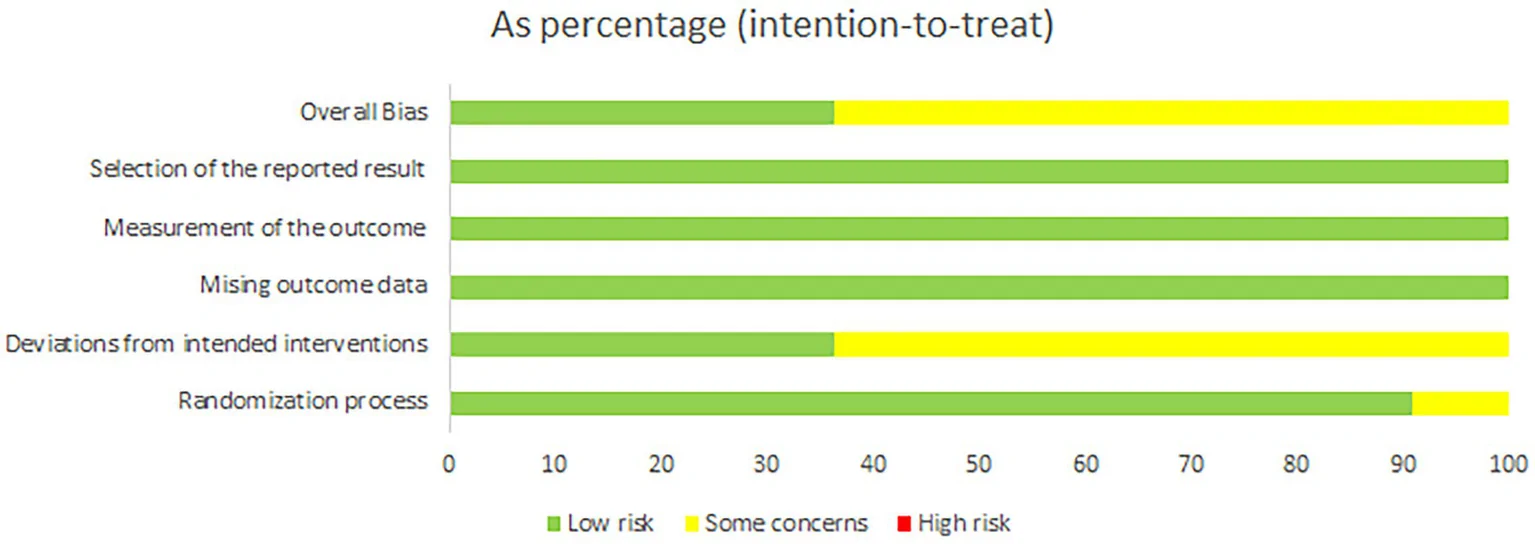
Risk of bias graph.

The risk of bias in the included studies was assessed using the Cochrane Risk of Bias Assessment Tool 2.0 (RoB 2.0). Overall, four studies were rated as having low risk of bias, and seven studies were rated as having some concerns. No studies were rated as having high risk of bias Figure 3.

Most studies showed low risk of bias in terms of missing outcome data, bias in measurement of the outcome, and bias in selection of the reported result. The most common potential source of bias was bias due to deviations from intended interventions, leading to seven studies being rated as having “some concerns.” additionally, one study raised some concerns with the randomization process. Overall, the risk of bias judgments suggested that the pooled estimates were unlikely to be substantially influenced by systematic bias (Figure 3 and Supplementary S3).

## Discussion

This systematic review and meta-analysis assessed the impact of motion-sensing games on balance and functional performance in older adults with chronic diseases residing in the community. Eleven randomized controlled trials involving 442 participants were included. Pooled results showed that, compared with control interventions, motion-sensing games significantly improved balance (assessed using the Berg Balance Scale [BBS]) and dynamic gait (assessed using the Dynamic Gait Index [DGI]). Subgroup analyses further indicated that Wii Fit-based interventions improved balance more effectively than Kinect Sports-based interventions; however, the differences between platforms were not statistically significant. Overall, these findings support motion-sensing games as a promising rehabilitation strategy for improving physical function in older adults with chronic diseases residing in the community.

The benefits observed in this review can be explained by several mechanisms. Motion-sensing games combine repetitive, task-oriented movements with real-time visual and auditory feedback, creating a rich training environment that promotes motor learning and postural control (13, 14). Many motion-sensing game projects involve multidirectional weight transfers, stepping, stretching, and balance challenges—activities highly similar to functional tasks in daily life (51). Such activities may provide repeated sensorimotor practice and task-oriented movement training, which could partly explain the observed improvements in balance and mobility; however, these underlying mechanisms were not directly assessed in the included studies (52, 53). Furthermore, the interactivity of motion-sensing games encourages cognitive engagement and active participation, which may contribute to improved functional performance; however, the specific mechanisms remain unclear (50, 54).

Another potential advantage of motion-sensing games is their ability to improve movement motivation and adherence (15). Traditional rehabilitation programs are often perceived as repetitive and monotonous, which may reduce long-term engagement in older adults. In contrast, game-based movement environments provide immediate feedback, achievable goals, and engaging challenges, thereby increasing engagement and training adherence. Improved adherence can lead to greater cumulative exercise volume, resulting in better functional recovery (8). Significant improvements in Dynamic Gait Index (DGI) scores are particularly noteworthy, as dynamic gait performance is closely associated with fall risk, activity limitations, and independence in older adults in the community (55).

The findings of this review are largely consistent with previous systematic reviews that have reported positive effects of motion-sensing games on physical function in older adults. However, most previous reviews have focused on specific populations, such as healthy older adults, institutionalized older adults, or individuals with neurological disorders. In contrast, this review specifically targets older adults living in the community with chronic diseases, a group facing a higher risk of functional decline and falls. By focusing on this population, this study expands the existing evidence base and demonstrates that motion-sensory games may benefit patients with a range of chronic diseases characterized by balance and mobility impairments. Although the underlying diseases involved in the studies varied, balance dysfunction and declining functional performance are common clinical challenges, highlighting the importance of integrating evidence from these populations.

These findings have significant clinical implications. Motion-sensory games offer a convenient and engaging adjunctive approach alongside traditional exercise interventions, particularly suitable for community rehabilitation programs (26). Because many motion-sensory game systems can be implemented in home, community center, and outpatient settings, they can promote sustained physical activity among older adults and support healthy aging at home (56). Furthermore, motion-sensory games may be especially valuable for those who lack motivation or find it difficult to adhere to traditional exercise programs. Therefore, rehabilitation professionals may consider incorporating motion-sensory games into multidisciplinary interventions aimed at improving balance, mobility, and fall prevention in older adults with chronic diseases.

This review has several strengths. First, by including only randomized controlled trials, it improves the overall quality of evidence. Second, the comprehensive search strategy across multiple databases reduces the possibility of missing relevant studies. Third, subgroup analyses based on motion-sensing game platforms provide further insights into potential differences in interventions.

However, there are also some limitations. The relatively small number of included studies and participants may limit statistical power. Significant heterogeneity exists in participant characteristics, chronic diseases, intervention protocols, and outcome measures. Furthermore, most studies assessed short-term outcomes, and evidence regarding the long-term effectiveness and sustainability of motion-sensing games remains limited. Finally, with fewer than 10 studies available for each pooled outcome, publication bias cannot be formally assessed.

Future research should include larger-scale, multicenter, randomized controlled trials using standardized intervention protocols and longer follow-up periods. Future studies should also investigate specific intervention parameters, including optimal training frequency, session duration, intervention length, exercise intensity, and level of supervision, to establish evidence-based prescriptions for motion-sensing game interventions. Further studies are needed to determine the most effective intervention characteristics for motion-sensory gaming interventions for older adults with chronic diseases. In addition, comparative studies are needed to determine whether effects differ across chronic disease populations, functional ability levels, and motion-sensing platforms. In addition, future research should evaluate long-term adherence, cost-effectiveness, feasibility, and the relative effectiveness of different motion-sensory gaming platforms in community settings.

## Conclusion

For older adults in the community with chronic diseases, motion-sensing games may represent a promising adjunctive intervention for improving their balance and functional performance. Significant improvements in balance and dynamic gait were observed in the motion-sensing game group compared to the control group. These findings support the potential incorporation of motion-sensing games as a complementary component of rehabilitation and community exercise programs. However, more high-quality, larger-sample, and longer-follow-up randomized controlled trials are needed to further strengthen the evidence base and determine the long-term clinical benefits of motion-sensing games.

## Data Availability

The original contributions presented in the study are included in the article/Supplementary material, further inquiries can be directed to the corresponding author.
