# Supplementary material for: Effects of exergaming on balance and functional performance in older adults with chronic conditions living in the community: a systematic review and meta-analysis
Source: Front Public Health. 2026 Sep 7;14:1918722. doi: 10.3389/fpubh.2026.1918722 (PMC13591622; doi:10.3389/fpubh.2026.1918722)
Supplement: Supplementary file 1 [file Table_1.docx]

**Supplementary Table 1 The PICOs details**

| **Participant** | **Intervention** | **Control** | **Outcomes** | **Studies** |
| --- | --- | --- | --- | --- |
| Community-dwelling older adults aged 60 years or older with chronic conditions, including but not limited to stroke, Parkinson’s disease, diabetes mellitus, osteoarthritis, cardiovascular disease, and other chronic diseases associated with impaired balance or functional mobility. | Exergaming-based interventions, including interactive video game–based exercise programs delivered through platforms such as Nintendo Wii, Wii Fit, Microsoft Kinect, virtual reality (VR), immersive or non-immersive VR systems, and other motion-based or gesture-based gaming technologies. Interventions could be supervised or unsupervised and conducted in community or home settings. | Usual care, health education, wait-list control, no intervention, or other non-exercise control conditions. | Primary outcomes:  Balance performance (e.g., Berg Balance Scale [BBS], postural control measures)  Functional mobility (e.g., Timed Up and Go test [TUG], Dynamic Gait Index [DGI])  Secondary outcomes:  Gait performance (e.g., gait speed, 6-Minute Walk Test [6MWT])  Lower-extremity muscle strength  Functional exercise capacity  Activities of daily living  Fall risk and fall-related outcomes | Randomized controlled trials (RCTs) published in English. |
