# Supplementary material for: Effects of exergaming on balance and functional performance in older adults with chronic conditions living in the community: a systematic review and meta-analysis
Source: Front Public Health. 2026 Sep 7;14:1918722. doi: 10.3389/fpubh.2026.1918722 (PMC13591622; doi:10.3389/fpubh.2026.1918722)
Supplement: Supplementary file 2 [file Table_2.docx]

| Outcomes (Measurements) | WMD 95% CI, I2 | P value |
| --- | --- | --- |
| Berg Balance Scale (BBS) | 2.93 [0.44 to 5.41], I² = 69.1% | P＜0.021* |
| Dynamic Gait Index (DGI) | 0.72 [0.09 to 1.35], I² = 0.0% | P＜0.025* |

**Supplementary Table 2 Overall effects**

**Note**: Difference *: p ＜ 0.05
