# Supplementary material for: Effects of exergaming on balance and functional performance in older adults with chronic conditions living in the community: a systematic review and meta-analysis
Source: Front Public Health. 2026 Sep 7;14:1918722. doi: 10.3389/fpubh.2026.1918722 (PMC13591622; doi:10.3389/fpubh.2026.1918722)
Supplement: Supplementary file 3 [file Table_3.docx]

**Supplementary Table 3 Risk of bias**

| **Study ID** | **Randomization process** | **Deviations from intended interventions** | **Missing outcome data** | **Measurement of the outcome** | **Selection of the reported result** | **Overall Bias** |
| --- | --- | --- | --- | --- | --- | --- |
| ChanWLS et al (2024, English, China) | Low | Some concerns | Low | Low | Low | Some concerns |
| Ribas et al (2017, English, Brazil) | Low | Some concerns | Low | Low | Low | Some concerns |
| Karasu et al (2018, English, Turkey) | Low | Some concerns | Low | Low | Low | Some concerns |
| Yu et al (2020, English, China) | Low | Some concerns | Low | Low | Low | Some concerns |
| BachaJMR et al (2018, English, Brazil) | Low | Low | Low | Low | Low | Low |
| Huang et al (2025, English, China) | Low | Low | Low | Low | Low | Low |
| Singh et al (2013, English, Malaysia) | Low | Some concerns | Low | Low | Low | Some concerns |
| Lee et al (2017, English, China) | Low | Some concerns | Low | Low | Low | Some concerns |
| Franco et al (2012, English, America | Some concerns | Some concerns | Low | Low | Low | Some concerns |
| Carcelén-FraileMDC et al (2024, English, Spain) | Low | Low | Low | Low | Low | Low |
| Morone et al (2016, English, Italy) | Low | Low | Low | Low | Low | Low |
