# Supplementary material for: Effects of exergaming on balance and functional performance in older adults with chronic conditions living in the community: a systematic review and meta-analysis
Source: Front Public Health. 2026 Sep 7;14:1918722. doi: 10.3389/fpubh.2026.1918722 (PMC13591622; doi:10.3389/fpubh.2026.1918722)
Supplement: Supplementary file 4 [file Table_4.docx]

**Supplementary Table 4 search strategy**

2026.4.10

PubMed: 404

Cochrane Library: 244

Embase: 325

CINAHL: 89

WEB OF SCIENCE: 147

PubMed：

("exergaming"[Title/Abstract] OR "active video game*"[Title/Abstract] OR "interactive video game*"[Title/Abstract]OR "video game-based exercise"[Title/Abstract]OR "motion-based game*"[Title/Abstract]OR "gesture-based game*"[Title/Abstract]OR "virtual reality"[Title/Abstract]OR VR[Title/Abstract]OR "virtual reality training"[Title/Abstract]OR "virtual reality exercise"[Title/Abstract]OR "immersive virtual reality"[Title/Abstract]OR "non-immersive virtual reality"[Title/Abstract]OR Wii[Title/Abstract] OR "Wii Fit"[Title/Abstract] OR Kinect[Title/Abstract] OR "Microsoft Kinect"[Title/Abstract])

AND
("Aged"[Mesh] OR elderly[Title/Abstract] OR "older adult*"[Title/Abstract] OR "community-dwelling"[Title/Abstract])
AND
("Stroke"[Mesh] OR "Parkinson Disease"[Mesh] OR stroke[Title/Abstract] OR "Parkinson*"[Title/Abstract] OR "chronic disease*"[Title/Abstract])
AND
("Postural Balance"[Mesh] OR balance[Title/Abstract] OR "postural control"[Title/Abstract] OR "Berg Balance Scale"[Title/Abstract] OR "Timed Up and Go"[Title/Abstract] OR TUG[Title/Abstract] OR gait[Title/Abstract] OR "gait speed"[Title/Abstract] OR "6-minute walk"[Title/Abstract] OR 6MWT[Title/Abstract] OR "Functional Reach"[Title/Abstract] OR "Sit-to-Stand"[Title/Abstract])
AND
(randomized controlled trial[Publication Type] OR randomized[Title/Abstract] OR randomly[Title/Abstract]))

Cochrane Library

((exergaming OR "active video game*" OR "interactive video game*" OR "video game-based exercise" OR "motion-based game*" OR "gesture-based game*" OR "virtual reality" OR VR OR "virtual reality training" OR "virtual reality exercise" OR "immersive virtual reality" OR "non-immersive virtual reality" OR Wii OR "Wii Fit" OR Kinect OR "Microsoft Kinect"):ti,ab,kw)

AND

(MeSH descriptor: [Aged] explode all trees

OR elderly:ti,ab,kw

OR "older adult*":ti,ab,kw

OR "community-dwelling":ti,ab,kw)

AND

(stroke OR Parkinson OR "chronic disease"):ti,ab,kw

AND

(MeSH descriptor: [Postural Balance] explode all trees

OR balance:ti,ab,kw

OR "postural control":ti,ab,kw

OR "Berg Balance Scale":ti,ab,kw

OR "Timed Up and Go":ti,ab,kw

OR TUG:ti,ab,kw

OR gait:ti,ab,kw

OR "gait speed":ti,ab,kw

OR "6-minute walk":ti,ab,kw

OR 6MWT:ti,ab,kw

OR "Functional Reach":ti,ab,kw

OR "Sit-to-Stand":ti,ab,kw)

Embase

('exergaming'/exp OR 'active video game*':ti,ab OR 'interactive video game*':ti,ab

OR 'video game based exercise':ti,ab OR 'motion based game*':ti,ab

OR 'gesture based game*':ti,ab OR 'virtual reality'/exp

OR 'virtual reality':ti,ab OR vr:ti,ab

OR 'virtual reality training':ti,ab OR 'virtual reality exercise':ti,ab

OR 'immersive virtual reality':ti,ab OR 'non immersive virtual reality':ti,ab

OR wii:ti,ab OR 'wii fit':ti,ab

OR kinect:ti,ab OR 'microsoft kinect':ti,ab

)

AND

(

'aged'/exp OR elderly:ti,ab OR 'older adult*':ti,ab OR 'community dwelling':ti,ab

)

AND

(

'stroke'/exp OR 'parkinson disease'/exp

OR stroke:ti,ab OR parkinson*:ti,ab

OR 'chronic disease*':ti,ab

)

AND

(

'balance'/exp OR 'postural balance'/exp

OR balance:ti,ab OR 'postural control':ti,ab

OR 'berg balance scale':ti,ab

OR 'timed up and go':ti,ab OR tug:ti,ab

OR gait:ti,ab OR 'gait speed':ti,ab

OR '6 minute walk':ti,ab OR 6mwt:ti,ab

OR 'functional reach':ti,ab

OR 'sit to stand':ti,ab

)

AND

(

'randomized controlled trial'/exp

OR random*:ti,ab

)

CINAHL

(

(MH "Virtual Reality+") OR (MH "Video Games+")

OR TI (exergaming OR "active video game*" OR "interactive video game*" OR "video game-based exercise" OR "motion-based game*" OR "gesture-based game*" OR "virtual reality" OR VR OR "virtual reality training" OR "virtual reality exercise" OR "immersive virtual reality" OR "non-immersive virtual reality" OR Wii OR "Wii Fit" OR Kinect OR "Microsoft Kinect")

OR AB (exergaming OR "active video game*" OR "interactive video game*" OR "video game-based exercise" OR "motion-based game*" OR "gesture-based game*" OR "virtual reality" OR VR OR "virtual reality training" OR "virtual reality exercise" OR "immersive virtual reality" OR "non-immersive virtual reality" OR Wii OR "Wii Fit" OR Kinect OR "Microsoft Kinect")

)

AND

(

(MH "Aged+")

OR TI (elderly OR "older adult*" OR "community-dwelling")

OR AB (elderly OR "older adult*" OR "community-dwelling")

)

AND

(

(MH "Stroke+") OR (MH "Parkinson Disease+")

OR TI (stroke OR Parkinson* OR "chronic disease*")

OR AB (stroke OR Parkinson* OR "chronic disease*")

)

AND

(

(MH "Postural Balance+")

OR TI (balance OR "postural control" OR "Berg Balance Scale" OR "Timed Up and Go" OR TUG OR gait OR "gait speed" OR "6-minute walk" OR 6MWT OR "Functional Reach" OR "Sit-to-Stand")

OR AB (balance OR "postural control" OR "Berg Balance Scale" OR "Timed Up and Go" OR TUG OR gait OR "gait speed" OR "6-minute walk" OR 6MWT OR "Functional Reach" OR "Sit-to-Stand")

)

AND

(

PT randomized controlled trial

OR TI (random* OR RCT)

OR AB (random* OR RCT)

)

WEB OF SCIENCE

TS=(

exergaming OR "active video game*" OR "interactive video game*"

OR "video game-based exercise" OR "motion-based game*"

OR "gesture-based game*" OR "virtual reality" OR VR

OR "virtual reality training" OR "virtual reality exercise"

OR "immersive virtual reality" OR "non-immersive virtual reality"

OR Wii OR "Wii Fit" OR Kinect OR "Microsoft Kinect"

)

AND TS=(

elderly OR "older adult*" OR "community-dwelling"

)

AND TS=(

stroke OR Parkinson* OR "chronic disease*"

)

AND TS=(

balance OR "postural control" OR "Berg Balance Scale"

OR "Timed Up and Go" OR TUG OR gait OR "gait speed"

OR "6-minute walk" OR 6MWT

OR "Functional Reach" OR "Sit-to-Stand"

)

AND TS=(

random* OR RCT

)
